# Synchronized modulation Kelvin probe force microscopy for surface photovoltage studies in optoelectronic systems

**DOI:** 10.1557/s43579-025-00899-3

**Published:** 2026-01-08

**Authors:** Zeinab Eftekhari, Ariane Ufer, Ursula Wurstbauer, Rebecca Saive

**Affiliations:** 1 MESA+ Institute for Nanotechnology, University of Twente, Enschede, The Netherlands; 2 Institute of Physics and Center for Soft Nanoscience (SON), University of Münster, Münster, Germany

**Keywords:** Scanning Probe Microscopy (SPM), Operando, Electronic material, Nanoelectronics, Optoelectronic, Nanostructure, Nanoscale, Photovoltaic

## Abstract

We introduce synchronized modulation Kelvin probe force microscopy (SM-KPFM), an in-operando technique that synchronizes external stimulus modulation, such as illumination or bias, with the scanning direction of the atomic force microscope to minimize artifacts from drift, thermal effects, and probe degradation. In synchronized illumination KPFM, the sample is illuminated during retrace and dark during trace, enabling real-time comparison of surface potentials within a single raster frame. At the example of a silicon photodiode and a molybdenum disulfide bilayer on gold, these superior properties are demonstrated, yielding drift-free, accurate surface photovoltage maps and clearer insights into photovoltaic effects.

**Graphical abstract:**

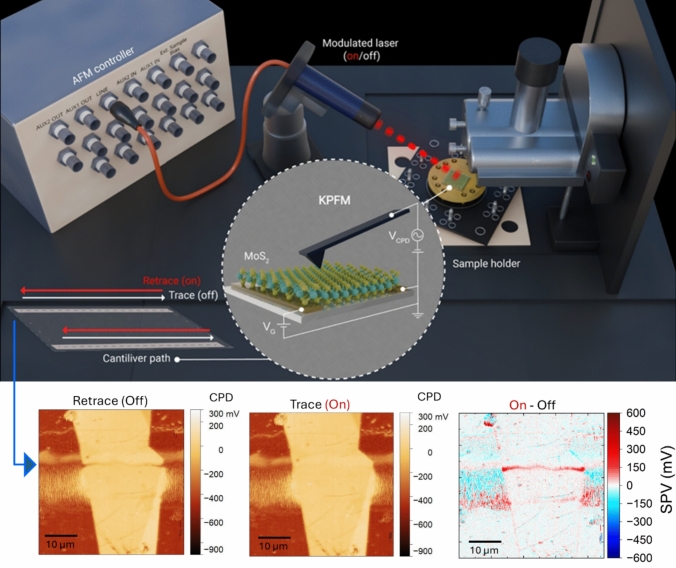

**Supplementary Information:**

The online version contains supplementary material available at 10.1557/s43579-025-00899-3.

Kelvin probe force microscopy (KPFM) is a versatile tool for nanoscale electrical characterization that enables spatial mapping of surface potential or work function. This is achieved by measuring the local contact potential difference (CPD) between a conductive scanning probe microscopy (SPM) tip and the sample surface.^[1,2]^ KPFM has presented itself as a powerful method for exploring the microscopic electric properties of semiconductors, 2D materials, and nanostructured interfaces.^[3]^ In particular, KPFM under illumination enables the mapping of surface photovoltage (SPV), defined as the difference between the CPD in the light and in the dark (SPV = CPD _light_–CPD _dark_),^[4,5]^ offering insight into charge separation and extraction mechanisms relevant to photovoltaic^[6,7]^ and other optoelectronic devices.^[8,9]^

Traditionally, SPV mapping by KPFM involves acquiring two sequential (partial) images, one in the dark and one under illumination.^[10–13]^ However, this approach is prone to spatial drift, tip wear, and thermal artifacts, which can reduce accuracy, particularly in materials with nanoscale heterogeneity. Time-resolved KPFM (tr-KPFM) addresses some of these challenges by modulating illumination or bias during acquisition to capture transient surface potential changes.^[14–17]^ However, tr-KPFM often requires synchronization hardware and signal averaging,^[18]^ which can increase experimental complexity and obscure localized variations in SPV, specially in materials with spatially varying surface properties. A related technique, nano-SPV, provides high-resolution, pixel-by-pixel data, capturing detailed illumination information for each pixel. While this offers powerful insights into charge dynamics, it generates large volumes of data, which may be excessive for routine SPV studies that don’t require such fine detail.^[19]^

In this study, we introduce synchronized modulation Kelvin probe force microscopy (SM-KPFM), a technique that synchronizes external stimulus modulation (e.g., illumination, bias or both) with the AFM scan direction. Specifically, we implement synchronized illumination KPFM, where the trace scan is performed in the dark and the retrace scan under illumination. This approach captures both CPD states along identical scan paths within a single raster frame and only requires triggering the light source with the AFM scan direction signal, ensuring precise spatial alignment and minimizing drift- and tip-related inconsistencies.

KPFM measurements were performed in sideband mode^[20]^ using a commercial SPM system (NX10, Park Systems Corp.) under ambient conditions. A Pt/Ir-coated silicon probe (ARROW-EFM, NanoWorld) with a resonance frequency of 68 kHz and a spring constant of 2.8 N/m was employed for high-sensitivity surface potential mapping.

For photoexcitation, a top-illuminated laser diode module (Premier PWM) operating at a wavelength of 635 nm and an output power of 5 mW was integrated into the AFM setup as shown in Fig. 1. The laser spot size of approximately 250 µm × 500 µm is considerably larger than the dimensions of the scanned area of the material systems studied in our paper. The laser modulation was synchronized directly with the scan direction using the system’s built-in Line Output, a standard BNC port on the AFM controller. During the trace scan, the Line Output gives a 0 V signal which kept the laser off (dark condition), while a 5 V signal during the retrace scan turned the laser on (illuminated condition). This direct hardware link enabled precise, real-time modulation of illumination without the need for external synchronization circuits or optical shutters.

**Figure 1:**
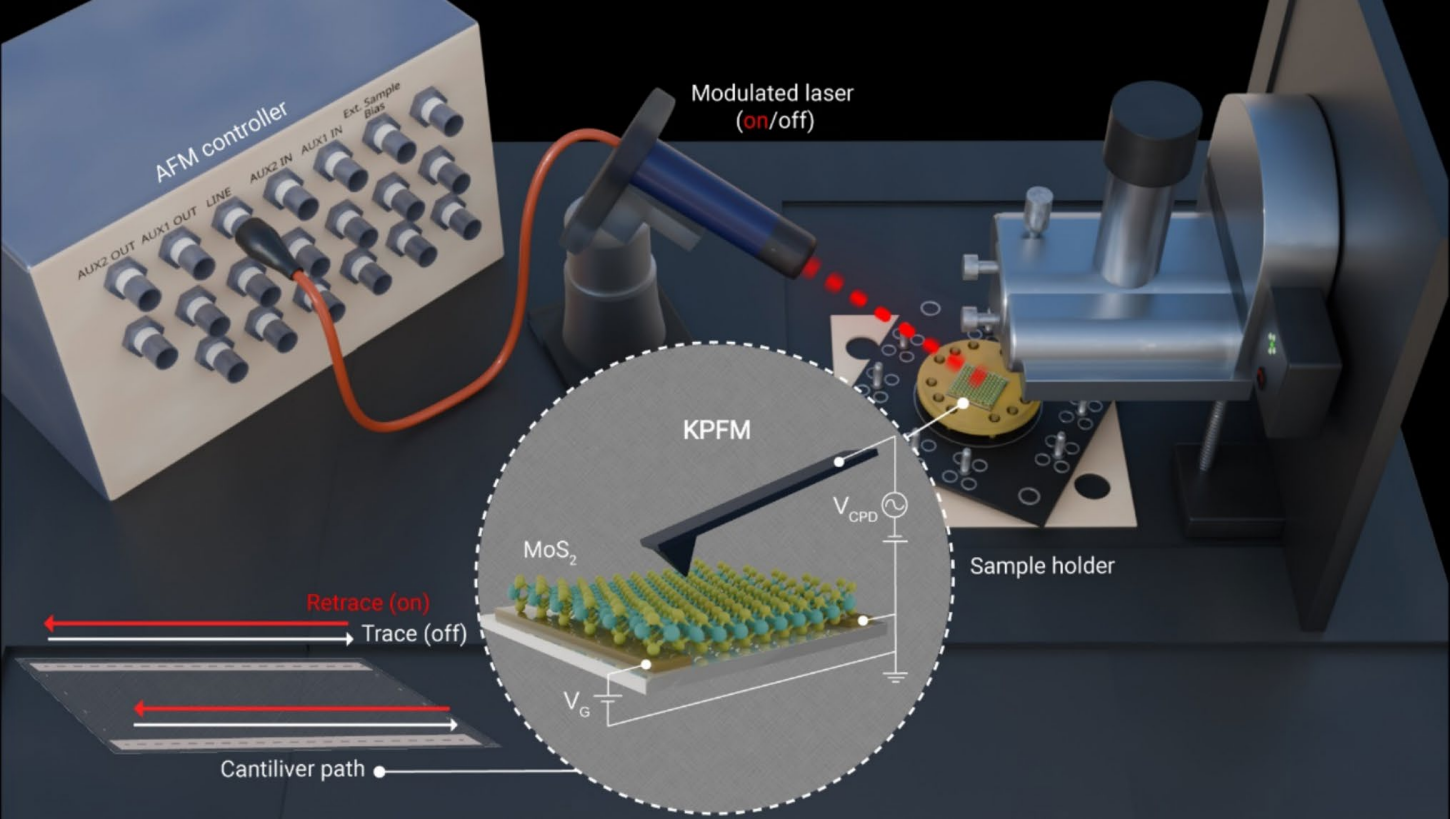
Schematic illustration of the synchronized Modulation Kelvin probe force microscopy (SM-KPFM) setup. In this method, external stimulus modulation, such as illumination and/or bias (V_G_), is synchronized with the AFM scan direction. A modulated laser (635 nm, 5 mW) is connected directly to the AFM controller’s Line Output, enabling real-time switching between dark (trace scan) and illuminated (retrace scan) conditions without external synchronization hardware. The cantilever path is showing the raster scanning of the sample, with the laser turned off during the trace scan and turned on during the retrace scan, producing two images: one for the trace in the dark and one for the retrace under illumination. The inset shows a representative MoS₂ sample on gold electrodes, with contact potential difference (V_CPD_) measured using a Pt/Ir-coated AFM tip.

AFM images were acquired in a standard raster-scanning mode: the tip scanned the sample line-by-line in the x-direction, with the trace and retrace acquired on the exact same line. After completing each line, the scanner incrementally advanced in the y-direction to generate a two-dimensional map. As a result, two spatially aligned datasets were collected simultaneously within a single scan, one under dark conditions (trace) and one under illumination (retrace). Post-processing involved pixel-wise subtraction of the retrace and trace images to quantify the change in surface potential upon illumination, reflecting the local charge redistribution and band bending effects induced by photoexcitation.

In parallel, subtraction of the topography images acquired during the trace and retrace passes was performed to monitor and quantify sample drift and thermal expansion effects. Because topography should ideally remain unchanged under illumination, any deviations in the on–off image serve as a direct indicator of mechanical or thermal instability during scanning.

We apply SM-KPFM to two systems: a commercial silicon photodiode and a photoabsorbing molybdenum disulfide (MoS₂) bilayer flake on gold electrodes. To benchmark the technique, we first measured a commercial silicon photodiode (FDS1010, Thorlabs). An area of 5 × 5 µm^2^ on the gold bar of the photodiode was selected for measurements. The region is conductive and, due to its surface texture, highlights drift effects more clearly in the topography images.

In the conventional method, separate scans were performed under dark and illuminated conditions. We acquired raw topography maps in the dark [Fig. 2(a)] and under illumination [Fig. 2(b)]. The topography difference map [Fig. 2(c)] was obtained by pixel-wise subtraction of the dark image from the illuminated image, revealing significant spatial drift. We post-processed the data in Gwyddion to correct spatial drift by registering the illuminated topography map to the dark reference, yet measurable spatial artifacts could not be fully removed (see supplementary material). In comparison, the SM-KPFM method records both dark and illuminated states within a single scan cycle. The raw topography images from the trace (dark) and retrace (illuminated) scans are shown in Fig. 2(d), (e), respectively. Figure 2(f) presents the topography difference acquired by subtracting the trace from retrace scan, confirming near-zero drift. To quantify the lateral misregistration between dark and illuminated frames, we calculated the root mean square deviation (RMSE) of the on–off images. For topography, where illumination should not change the surface, any signal from the topography difference image is drift. Quantitatively, the RMSE decreases from 35.8 nm in the conventional method to 20.0 nm after drift correction (a 44% reduction), and further to 1.11 nm with SM-KPFM acquisition (a 97% reduction relative to conventional and 94% relative to drift-corrected). These reductions indicate that synchronized imaging nearly eliminates inter-frame misregistration.

**Figure 2:**
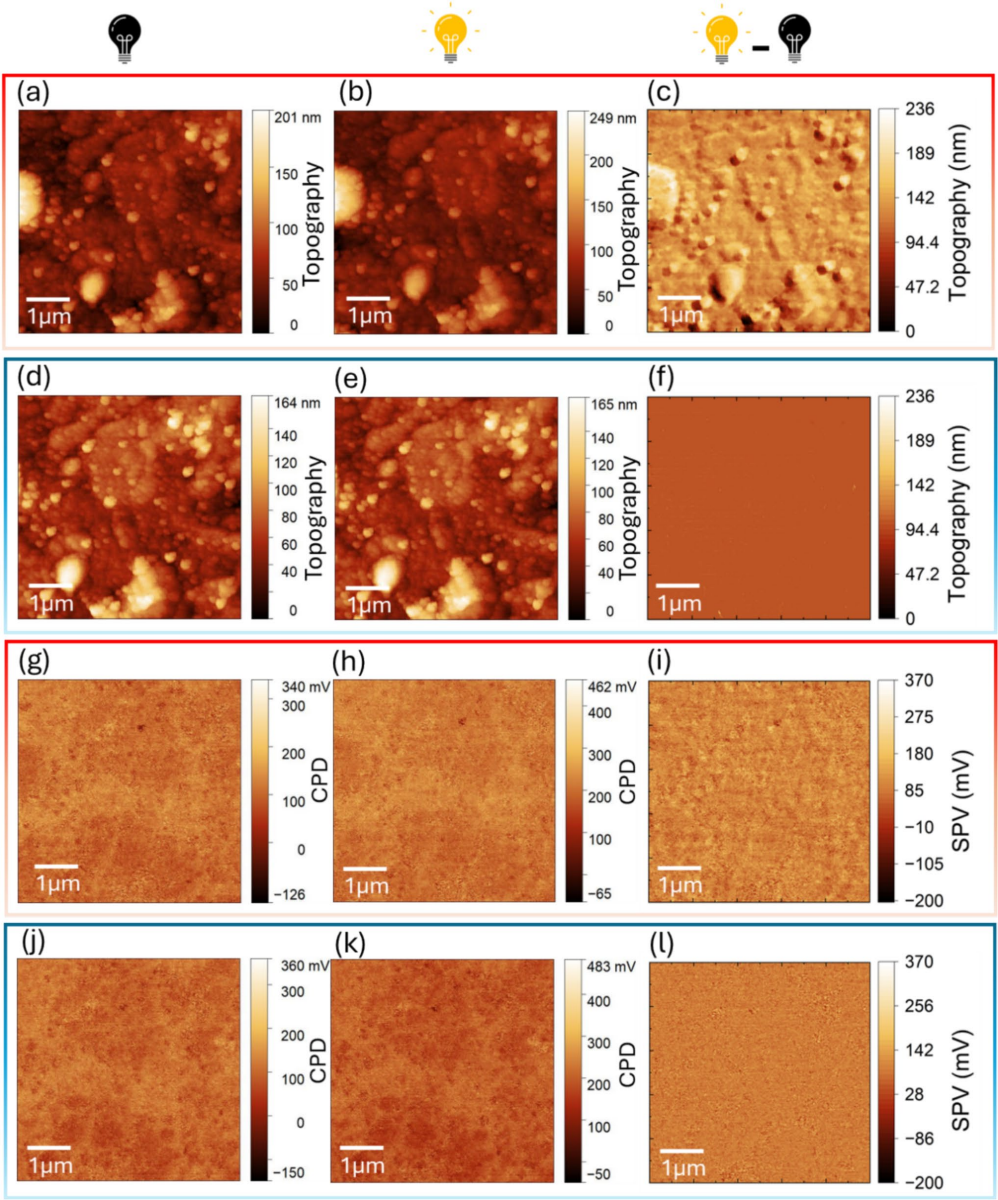
Comparison of surface topography and surface potential measurements of a silicon photodiode using conventional (a–c, g–i) (red box) and synchronized illumination KPFM (d–f, j–l) (blue box). (a, b) Topography acquired in two separate scans in the dark (a) and under illumination (b). (c) Topography difference image showing spatial drift and misalignment artifacts. (d, e) Topography during trace (dark (d)) and retrace (illuminated (e)) in a single synchronized scan. (f) Topography difference image (trace-retrace) showing minimal drift. (g, h) Surface potential from separate dark and illuminated scans. (i) Resulting SPV map exhibiting artifacts and inhomogeneities. (j, k) Surface potential during trace and retrace in synchronized scan. (l) SPV map with high uniformity and minimal artifacts, highlighting the advantage of synchronized KPFM.

The corresponding CPD images in the conventional KPFM were taken in the dark [Fig. 2(g)] and under illumination condition [Fig. 2(h)] in sequential acquisitions. Figure 2(i) shows the SPV map derived by subtracting the dark scan image from the illuminated one, featuring clear non-uniformities. In SM-KPFM, the surface potential maps in the dark are obtained in trace [Fig. 2(j)] and under illumination in retrace [Fig. 2(k)] scanning direction. The resulting SPV map [Fig. 2(l)] demonstrates a much more homogeneous photovoltage distribution, indicating reliable detection of photo-induced charge redistribution. Unlike topography, the SPV map cannot be judged by absolute RMSE because it contains both genuine photovoltage and residual drift. To isolate the drift contribution, we take the SM-KPFM SPV as a drift-minimized baseline and, assuming weak correlation between SPV and drift, estimate the drift power in the MSE domain as MSE_drift_ ≈ MSE_method_ − MSE_SM-KPFM_. With MSE = 0.01201 V^2^ (conventional), 0.01170 V^2^ (drift-corrected), and 0.01007 V^2^ (SM-KPFM), we obtain MSE_drift_ = 1.94 × 10⁻^3^ V^2^ for conventional and 1.63 × 10⁻^3^ V^2^ for the drift-corrected map, corresponding to RMSE_drift_ ≈ 44.0 mV and 40.4 mV, respectively, indicating a 16% reduction in drift power with registration.Accordingly, the remaining signal in the SPV map of SM-KPFM is dominated by genuine SPV, with only minor drift contamination. This comparison highlights the enhanced reproducibility and stability of SM-KPFM, particularly when analyzing spatially nonuniform or sensitive materials.

To further demonstrate the broader applicability of SM-KPFM beyond a well-established reference system, we investigated molybdenum disulfide (MoS₂). As an emerging optoelectronic material, MoS₂ combines exceptional electronic and optical properties with many open questions regarding its nanoscale photoresponse. Its strong light absorption and pronounced behavior at metal–semiconductor junctions make it a highly relevant platform for studying spatially and temporally resolved SPV phenomena. As a case study, we examined a bilayer MoS₂ flake transferred onto gold electrodes on a glass substrate. The one-nanometer-thick indirect semiconductor was isolated from bulk crystals by micromechanical exfoliation and transferred via viscoelastic stamping. The flake bridges a 15 µm channel between two gold electrodes with a height of 30 nm. Its geometry and successful transfer are shown in the topography and optical images in Fig. 3(a), (b).

**Figure 3:**
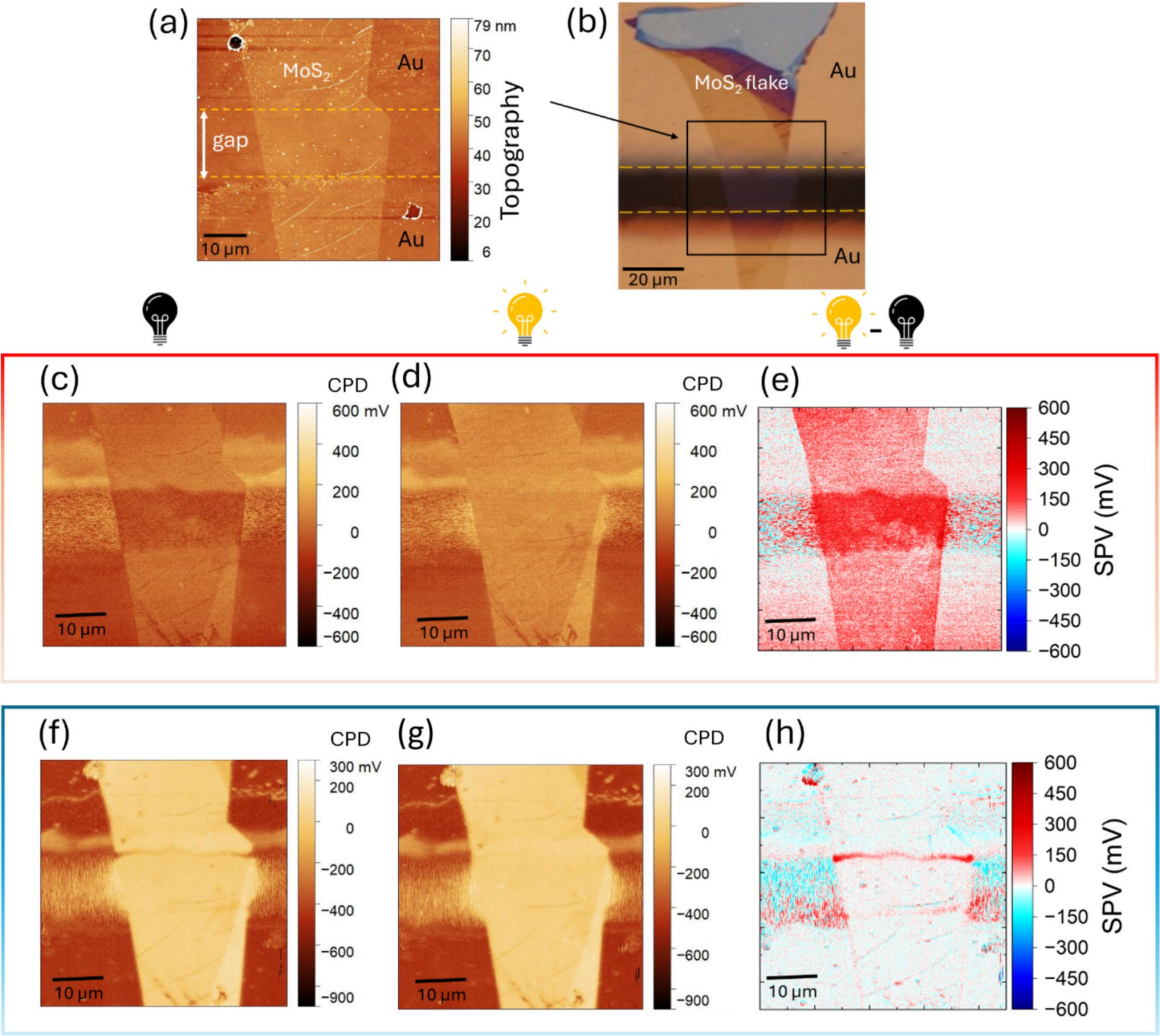
KPFM measurements of a MoS₂ flake on gold electrodes under dark and illuminated conditions. (a) Topography and (b) optical image of the MoS₂ flake on the gold electrodes, where the black box shows the scanned area under AFM/KPFM. The topography image was post-processed to have the substrate and gold contact surfaces on the same level such that the thin flake becomes visible. (c, d) CPD maps acquired in separate scans under dark (c) and illuminated (d) conditions using conventional KPFM (red box). (e) SPV map derived from the sequential scans. (f) Trace (dark) and (g) retrace (illuminated) CPD maps obtained using SM- KPFM (blue box). (h) SPV map (retraced minus trace).

Here we compare surface potential measurements of the MoS₂ sample using the two different KPFM methods: conventional sequential KPFM and SM- KPFM. The scan area was set to 45 × 45 µm^2^, corresponding to the dimensions of the investigated flake. At a scan rate of 0.2 Hz with 512 × 512 pixels, each image required ~ 42 min to acquire, with a pixel dwell time of ~ 10 ms.

In conventional KPFM, separate scans were taken to measure CPD of the MoS2 sample under dark [Fig. 3(c)] and illuminated [Fig. 3(d)] conditions. We take the bare Au electrodes as the reference, and the CPD of the MoS₂ flake is compared relative to this baseline. In the dark scan, the bilayer MoS₂ on the Au electrodes appears ≈ 200 mV higher in CPD than bare Au which arises from their work function difference, whereas within the channel (MoS₂ on glass), the potential of MoS₂ is effectively the same as Au. This can be attributed to the charge carriers trapped at the MoS₂-glass interface which depletes the flake and shifts its Fermi level downward, thereby suppressing the CPD contrast relative to Au. Under illumination, the entire flake shifts uniformly to ≈ 250 mV above Au, including the region spanning the channel which can be explained by a screening effect or detrapping of charge carriers. The subtraction of the dark scan from the illuminated one is shown in Fig. 3(e) representing the SPV map. It exhibits strong photovoltage signal across the entire MoS₂ flake, with an even more pronounced signal within the electrode gap.

In the SM- KPFM technique, the CPD maps for the dark (trace) and illuminated (retrace) states are shown in Fig. 3(f), (j), respectively. The CPD map acquired in the dark reveals that the MoS₂ flake exhibits a surface potential approximately 450 mV higher than the bare Au electrodes, except at the edges of the contacts within the channel, where the potential locally collapses toward the Au value. Under illumination, the CPD of the entire flake becomes uniformly shifted by about 450 mV relative to Au, including the regions at the edged in the channel. The SPV map (retrace minus trace) in Fig. 3(h) shows a contrast localized sharply at the edges of the MoS₂ flake near the metal contacts within the channel.

The distinct contrasts of the resultant SPV maps acquired by conventional and SM-KPFM methods arise from a combination of intrinsic photovoltaic responses and slower extrinsic effects. When the MoS₂ sample is illuminated, several mechanisms can contribute to the measured photoresponse. The primary photovoltaic effect originates from electron–hole pairs generated in MoS₂ and separated by the built-in electric fields at the MoS₂-Au Schottky barriers.^[21–26]^ This process produces a short-circuit current and open-circuit voltage, with carrier generation and separation occurring on ultrafast ps–ns scales, while diffusion and recombination across the channel extend into the µs–ms range. In addition to this intrinsic photovoltaic contribution, photogating can arise when one type of carrier, typically holes, becomes trapped at the MoS₂/substrate interface. The resulting trapped charges act as a local gate, modifying channel conductivity and producing a persistent response that evolves on much slower timescales from microseconds to seconds.^[27]^ Even slower contributions include thermally induced potentials such as photothermal or ionic charging effects, which evolve over seconds to minutes. Laser-induced heating can alter the local potential via thermal drift, particularly in MoS₂, which has low thermal conductivity.^[28,29]^ Because the sample is illuminated globally, both contacts and the flake are heated uniformly, therefore the photothermoelectric effect is negligible.

Due to its sequential scan acquisition, conventional KPFM integrates signals across the full range of timescales. The long delay between dark and illuminated images allows sufficient time for slow processes like photogating, residual charging at the MoS₂–glass interface, and laser-induced heating to develop, leading to strong, spatially extended SPV signals across the flake and in the channel. SM-KPFM, by contrast, alternates dark and illuminated states line-by-line, with a delay of only tens of milliseconds. On this timescale, fast electronic processes such as photovoltaic separation and interfacial carrier redistribution are expressed, whereas slower trap-mediated and thermal contributions are largely suppressed. Although, SM-KPFM cannot resolve ultrafast carrier dynamics, it is fast enough to capture the net SPV shaped by these processes, while remaining sensitive to electronic and interfacial responses that evolve on the µs–ms timescale. Consequently, SM-KPFM highlights the localized interfacial SPV at MoS_2_–Au contacts, while conventional KPFM produces broadened maps that convolve both the intrinsic photovoltaic effect and slower extrinsic mechanisms. The difference between the dark states recorded by each method arises primarily from the different temporal windows each technique probes.

In addition, we investigated the effect of scan direction on the observed SPV in the MoS₂ sample using synchronized illumination KPFM. The measurement results of this study are shown in the supplemental material. While measuring with the scan direction across the MoS₂ flake, we detected noticeable edge effects in the SPV map. To further explore this, we varied the scan rates and found that the SPV at the edges became stronger and more pronounced with increasing the scan rate. However, when we rotated the scan direction by 90 degrees and scanned along the flake, no such effects were observed (see Supplementary Material). These edge effects can be attributed to time delays in the KPFM feedback controller when the tip passes over the gold/flake interface. These delays occur due to the time required for the KPFM feedback to adjust the tip’s position or voltage in response to changes in surface potential which can be more noticeable when scanning rapidly near material interfaces or edges.^[30]^ They lead to small misalignment between the actual surface features and the recorded surface potential, resulting in artifacts when subtracting two images that are taken with opposite scan direction. Therefore, caution is necessary when interpreting SPV results near edges, as these artifacts can lead to misinterpretations if not properly accounted for.

This work introduces synchronized modulation Kelvin probe force microscopy (SM-KPFM) as a powerful technique that overcomes the limitations of conventional sequential KPFM in surface potential mapping. By synchronizing light modulation with the scanning direction of AFM, SM-KPFM ensures that data are collected in a spatially aligned manner, effectively minimizing thermal and drift-induced artifacts that commonly compromise measurement accuracy. Our results from studying a silicon photodiode and semiconducting MoS₂ on gold electrodes demonstrate that SM-KPFM can reveal SPV distributions with high fidelity, offering insights into photoinduced charge dynamics that were previously obscured by drift distortion in conventional methods. Notably, SM-KPFM provides a more reliable and consistent SPV map without the complications associated with sequential scanning. The ability to measure steady-state SPV with precision in a single scan cycle positions SM-KPFM as an essential tool for nanoscale optoelectronic characterization. Looking forward, the versatility of SM-KPFM offers significant potential for exploring a wide range of 2D materials, their interfaces, and other thin film photo-absorbers on metal contacts, including organic materials that are prone to nanoscale topographical inhomogeneities. By applying a bias across electrodes while simultaneously modulating light, this technique can provide detailed insights into the material’s response to both electrical and optical stimuli. For instance, applying forward and reverse bias in combination with light illumination could reveal essential information about charge injection, separation, recombination, and transport at the material interfaces. This approach is not limited to MoS₂ but can be generalized to other TMDs, 2D materials and photo-sensitive thin-films, making SM-KPFM a valuable tool for advancing our understanding of optoelectronic properties and guiding the development of high-performance devices, such as photodetectors, solar cells, and transistors.

## Supplementary Information

Below is the link to the electronic supplementary material.

Supplementary file1 (DOCX 4895 KB)—See the supplementary material for the post processing treatment done in order to correct the drift for the conventional KPFM data and the investigation of scan direction affecting the SPV at the edges of MoS_2_ flake.

## Data Availability

The data that support the findings of this study are available from the corresponding author upon reasonable request.

## References

[1] M. Nonnenmacher, M.P. O’Boyle, H.K. Wickramasinghe, Kelvin probe force microscopy. Appl. Phys. Lett. **58**(25), 2921–2923 (1991)

[2] Y. Rosenwaks, R. Shikler, T. Glatzel, S. Sadewasser, Kelvin probe force microscopy of semiconductor surface defects. Phys. Rev. B Condens. Matter Mater. Phys. **70**(8), 085320 (2004)

[3] D. Moore, K. Jo, C. Nguyen, J. Lou, C. Muratore, D. Jariwala, N.R. Glavin, Uncovering topographically hidden features in 2D MoSe2 with correlated potential and optical nanoprobes. NPJ 2D Mater Appl **4**(1), 1–7 (2020)

[4] Z. Schumacher, Y. Miyahara, A. Spielhofer, P. Grutter, “Measurement of surface photovoltage by atomic force microscopy under pulsed illumination. Phys. Rev. Appl. **5**, 044018 (2016)

[5] P. González-Izquierdo, N. Rochat, M. Charles, T. Sochacki, Ł Borowik, Kelvin probe force microscopy under variable illumination: a novel technique to unveil charge carrier dynamics in GaN. J. Phys. Chem. C **127**(26), 12727–12734 (2023)

[6] R. Saive, C. Mueller, J. Schinke, R. Lovrincic, W. Kowalsky, Understanding S-shaped current-voltage characteristics of organic solar cells: direct measurement of potential distributions by scanning Kelvin probe. Appl. Phys. Lett. **103**(24), 243303 (2013)

[7] R. Saive, M. Scherer, C. Mueller, D. Daume, J. Schinke, M. Kroeger, W. Kowalsky, Imaging the electric potential within organic solar cells. Adv. Funct. Mater. **23**(47), 5854–5860 (2013)

[8] S.A.L. Weber, I.M. Hermes, S.H. Turren-Cruz, C. Gort, V.W. Bergmann, L. Gilson, A. Hagfeldt, M. Graetzel, W. Tress, R. Berger, How the formation of interfacial charge causes hysteresis in perovskite solar cells. Energy Environ. Sci. **11**(9), 2404–2413 (2018)

[9] C.S. Weigel, W. Kowalsky, R. Saive, Direct observation of the potential distribution within organic light emitting diodes under operation. Phys. Status Solidi (RRL) **9**(8), 475–479 (2015)

[10] W. Yim, V.T. Nguyen, Q.T. Phung, H.S. Kim, Y.H. Ahn, S. Lee, J.Y. Park, Imaging spatial distribution of photogenerated carriers in monolayer MoS2with kelvin probe force microscopy. ACS Appl. Mater. Interfaces **14**(22), 26295–26302 (2022) 35613454 10.1021/acsami.2c06315

[11] E.M. Tennyson, J.L. Garrett, J.A. Frantz, J.D. Myers, R.Y. Bekele, J.S. Sanghera, J.N. Munday, M.S. Leite, Nanoimaging of open-circuit voltage in photovoltaic devices. Adv. Energy Mater. **5**, 1501142 (2015)

[12] M. Vishwakarma, D. Varandani, C. Andres, Y.E. Romanyuk, S.G. Haass, A.N. Tiwari, B.R. Mehta, A direct measurement of higher photovoltage at grain boundaries in CdS/ CZTSe solar cells using KPFM technique. Sol. Energy Mater. Sol. Cells **183**, 34–40 (2018)

[13] R. Saive, Investigation of the Potential Distribution within Organic Solar Cells by Scanning Kelvin Probe Microscopy (Doctoral Dissertation), 2014

[14] Y. Almadori, N. Bendiab, B. Grévin, Multimodal kelvin probe force microscopy investigations of a photovoltaic WSe2/MoS2 type-II interface. ACS Appl. Mater. Interfaces **10**(1), 1363–1373 (2018) 29218991 10.1021/acsami.7b14616

[15] D.C. Coffey, D.S. Ginger, Time-resolved electrostatic force microscopy of polymer solar cells. Nat. Mater. **5**(9), 735–740 (2006) 16906141 10.1038/nmat1712

[16] J. Sato, R. Ishibashi, T. Takahashi, Time-resolved electrostatic force microscopy under base-bias-level control. Meas. Sci. Technol. **35**(3), 035005 (2023)

[17] Z. Eftekhari, N. Rezaei, H. Stokkel, J.Y. Zheng, A. Cerreta, I. Hermes, M. Nguyen, G. Rijnders, R. Saive, Spatial mapping of photovoltage and light-induced displacement of on-chip coupled piezo/photodiodes by Kelvin probe force microscopy under modulated illumination. Beilstein J. Nanotechnol. **14**(1), 1059–1067 (2023) 38025201 10.3762/bjnano.14.87 PMC10644008

[18] S. Sadewasser, N. Nicoara, S.D. Solares, Artifacts in time-resolved Kelvin probe force microscopy. Beilstein J. Nanotechnol. **9**(1), 1272 (2018) 29765805 10.3762/bjnano.9.119 PMC5942368

[19] Y. Yalcinkaya, P.N. Rohrbeck, E.R. Schütz, A. Fakharuddin, L. Schmidt-Mende, S.A.L. Weber, Nanoscale surface photovoltage spectroscopy. Adv. Opt. Mater. (2024)

[20] A. Axt, I.M. Hermes, V.W. Bergmann, N. Tausendpfund, S.A.L. Weber, Know your full potential: Quantitative Kelvin probe force microscopy on nanoscale electrical devices. Beilstein J. Nanotechnol. **9**(1), 1809–1819 (2018) 29977714 10.3762/bjnano.9.172 PMC6009372

[21] A. Abnavi, R. Ahmadi, H. Ghanbari, M. Fawzy, A. Hasani, T. De Silva, A.M. Askar, M.R. Mohammadzadeh, F. Kabir, M. Whitwick, M. Beaudoin, S.K. O’Leary, M.M. Adachi, Flexible high-performance photovoltaic devices based on 2D MoS2 diodes with geometrically asymmetric contact areas. Adv. Funct. Mater. **33**(7), 2210619 (2023)

[22] P.A. Markeev, E. Najafidehaghani, Z. Gan, K. Sotthewes, A. George, A. Turchanin, M.P. de Jong, Energy-level alignment at interfaces between transition-metal dichalcogenide monolayers and metal electrodes studied with kelvin probe force microscopy. J. Phys. Chem. C **125**(24), 13551–13559 (2021) 10.1021/acs.jpcc.1c01612 PMC8237262 34239657

[23] H. Wang, Z. Li, D. Li, X. Xu, P. Chen, L. Pi, X. Zhou, T. Zhai, Junction field-effect transistors based on PdSe2/MoS2 heterostructures for photodetectors showing high responsivity and detectivity. Adv. Funct. Mater. **31**(49), 2106105 (2021)

[24] M. Fontana, T. Deppe, A.K. Boyd, M. Rinzan, A.Y. Liu, M. Paranjape, P. Barbara, Electron-hole transport and photovoltaic effect in gated MoS2 Schottky junctions. Sci. Rep. **3**(1), 1–6 (2013) 10.1038/srep01634 PMC3620663 23567328

[25] E. Parzinger, M. Hetzl, U. Wurstbauer, A.W. Holleitner, Contact morphology and revisited photocurrent dynamics in monolayer MoS2. Npj 2D Mater. Appl. **1**(1), 1–8 (2017)

[26] S. Subramanian, Q.T. Campbell, S.K. Moser, J. Kiemle, P. Zimmermann, P. Seifert, F. Sigger, D. Sharma, H. Al-Sadeg, M. Labella, D. Waters, R.M. Feenstra, R.J. Koch, C. Jozwiak, A. Bostwick, E. Rotenberg, I. Dabo, A.W. Holleitner, T.E. Beechem, U. Wurstbauer, J.A. Robinson, Photophysics and electronic structure of lateral graphene/MoS2and metal/MoS2junctions. ACS Nano **14**(12), 16663–16671 (2020) 33196167 10.1021/acsnano.0c02527

[27] A. Di Bartolomeo, L. Genovese, T. Foller, F. Giubileo, G. Luongo, L. Croin, S.J. Liang, L.K. Ang, M. Schleberger, Electrical transport and persistent photoconductivity in monolayer MoS2 phototransistors. Nanotechnology **28**(21), 214002 (2017) 28471746 10.1088/1361-6528/aa6d98

[28] Y. Park, H.W. Baac, J. Heo, G. Yoo, Thermally activated trap charges responsible for hysteresis in multilayer MoS2 field-effect transistors. Appl. Phys. Lett. **108**(8), 48 (2016)

[29] J. Klein, A. Kerelsky, M. Lorke, M. Florian, F. Sigger, J. Kiemle, M.C. Reuter, T. Taniguchi, K. Watanabe, J.J. Finley, A.N. Pasupathy, A.W. Holleitner, F.M. Ross, U. Wurstbauer, Impact of substrate induced band tail states on the electronic and optical properties of MoS2. Appl. Phys. Lett. **115**(26), 261603 (2019)

[30] D.C. Coffey, D.S. Ginger, Time-resolved electrostatic force microscopy of polymer solar cells. Nat. Mater. (2006) 10.1038/nmat1712 16906141

