# Supplementary material for: Synchronized modulation Kelvin probe force microscopy for surface photovoltage studies in optoelectronic systems
Source: MRS Commun. 2026 Jan 8;16(1):180–6. doi: 10.1557/s43579-025-00899-3 (PMC13002633; doi:10.1557/s43579-025-00899-3)
Supplement: Supplementary file 1 — Supplementary file1 (DOCX 4895 KB)—See the supplementary material for the post processing treatment done in order to correct the drift for the conventional KPFM data and the investigation of scan direction affecting the SPV at the edges of MoS2 flake. [file 43579_2025_899_MOESM1_ESM.docx]

***Supplementary Information***

**Synchronized Modulation Kelvin Probe Force Microscopy for Surface Photovoltage Studies in Optoelectronic Systems**

Zeinab Eftekhari^1^, Ariane Ufer^2^, Ursula Wurstbauer^2^, Rebecca Saive^*1^

*^1^MESA+ Institute for Nanotechnology, University of Twente, The Netherlands*

*^2^Institute of Physics and Center for Soft Nanoscience (SON), University of Münster, Münster, Germany*

^*^Corresponding Author Email: [r.saive@utwente.nl](mailto:r.saive@utwente.nl),

**Post-processing of topography and surface potential data obtained by conventional KPFM for drift correction and comparing them to the results from SM-KPFM**

In the conventional KPFM approach, two separate scans were conducted, one in the dark and one under illumination. Topography maps from both the dark (Fig. S1(a)) and illuminated (Fig. S1(b)) conditions were used to calculate the topography difference by subtracting the dark map from the illuminated one (Fig. S1(c)). Subsequently, we applied post-processing treatment using Gwyddion to correct the spatial drift by aligning the topography map under illumination with the dark condition. The corrected image is shown in Fig. S1(e), and the topography difference (Fig. S1(f)) was calculated. However, even after applying drift correction, some noticeable spatial drift remains, which could not be fully eliminated through post-processing.


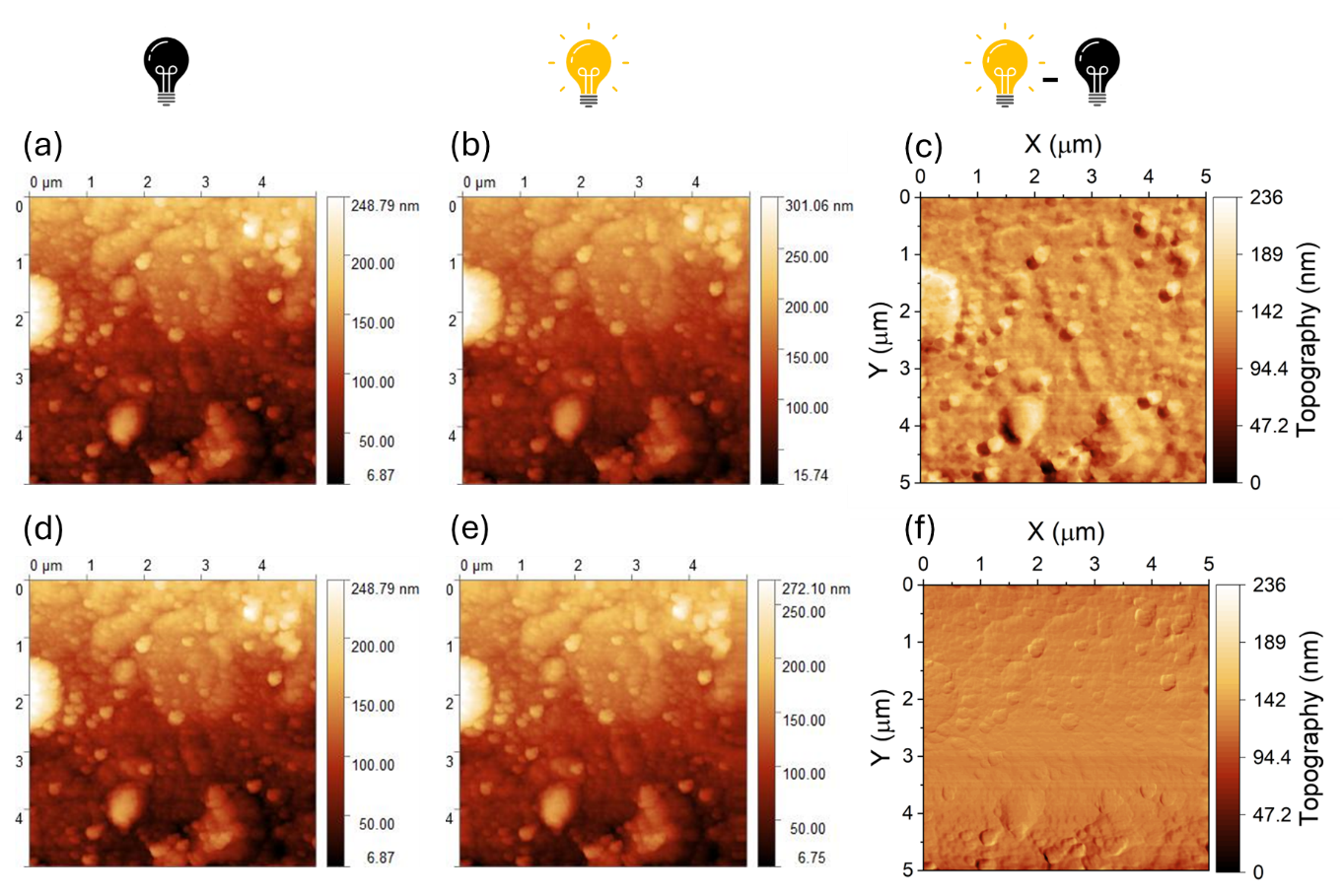


**FIG. S1**. Surface Topography of a Silicon Photodiode Using Conventional KPFM

(a–b) Topography images acquired in separate scans under dark and illuminated conditions.

(c) Topography difference image showing spatial drift and misalignment artifacts.

(d) Topography under dark conditions.

(e) Topography under illuminated conditions after drift correction, aligned to the dark image.

(f) Topography difference between the corrected illuminated and dark images.

Figure S2 presents the topography difference of the data acquired from conventional KPFM before (Fig. S2(a)) and after post-processing treatment (Fig. S2(b)), alongside the results from SM-KPFM (Fig. S2(c)). Despite the drift correction, the conventional KPFM data still exhibit spatial drift and misalignment, whereas SM-KPFM shows no drift in the topography difference. The SPV maps of conventional KPFM before (Fig. S2(d)) and after drift correction (Fig. S2(e)), as well as the SPV map from SM-KPFM (Fig. S2(f)), are shown. Even after drift correction, the conventional KPFM SPV map remains inhomogeneous, whereas the SM-KPFM map demonstrates superior uniformity. This comparison highlights that SM-KPFM provides more reliable SPV measurements, even when post-processing treatments are applied to conventional KPFM data.


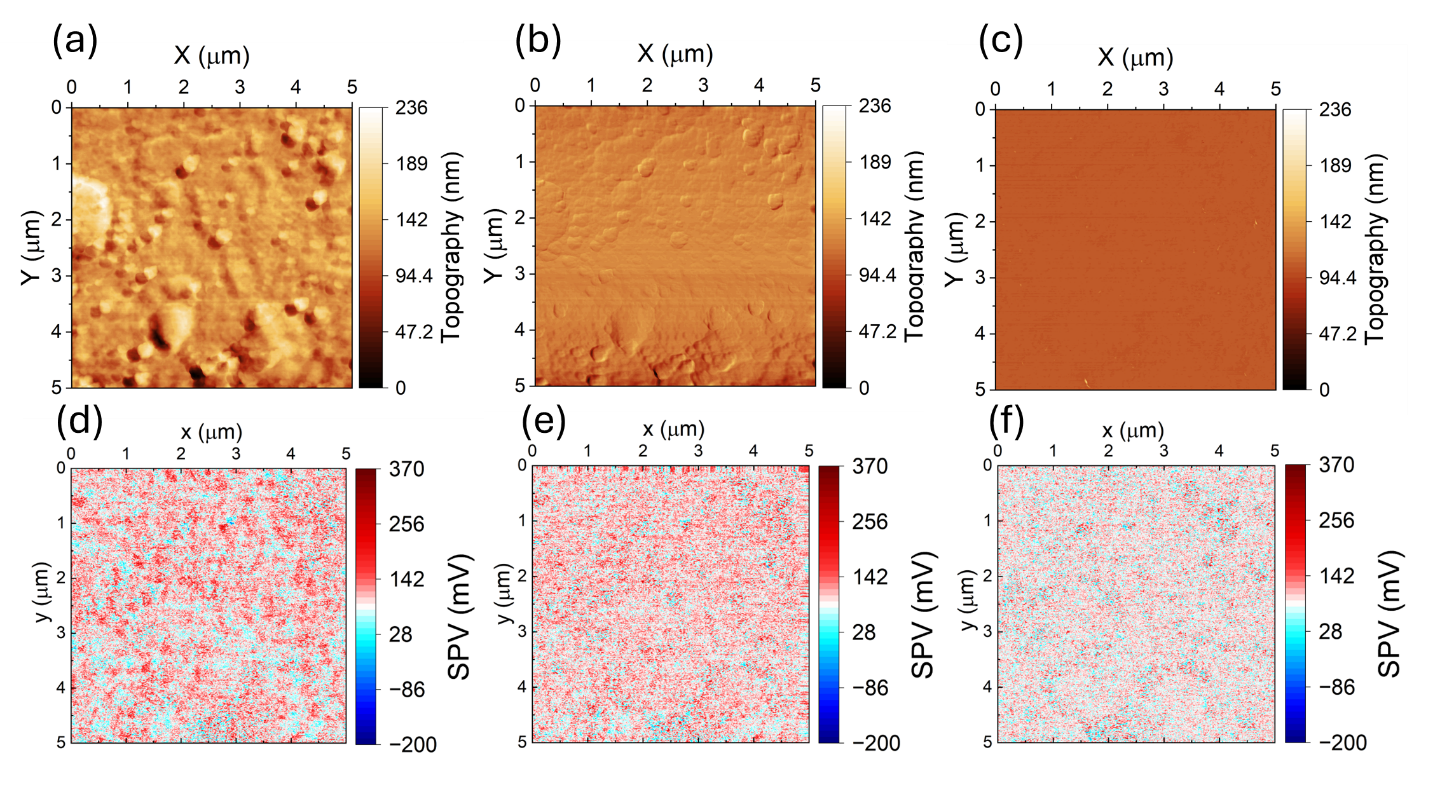


**FIG. S2.** Comparison of topography difference and SPV maps of a silicon photodiode obtained using conventional KPFM before and after drift correction, alongside SM-KPFM results.
(a) Topography difference map acquired using conventional KPFM.
(b) Topography difference map after drift correction applied to the conventional KPFM data.
(c) Trace-retrace topography difference map obtained using SM-KPFM, showing minimal drift.
(d) SPV map obtained from conventional KPFM.
(e) SPV map from conventional KPFM after drift correction.
(f) SPV map obtained using SM-KPFM.

**Scan direction in SM-KPFM**

The impact of scan direction on the observed SPV in the MoS₂ sample using synchronized illumination KPFM was examined. When scanning across the MoS₂ flake shown in Fig.S3(a), we observed significant edge effects in the SPV map. However, when we rotated the scan direction by 90 degrees to scan along the flake shown in Fig S3 (b), these effects were no longer present.


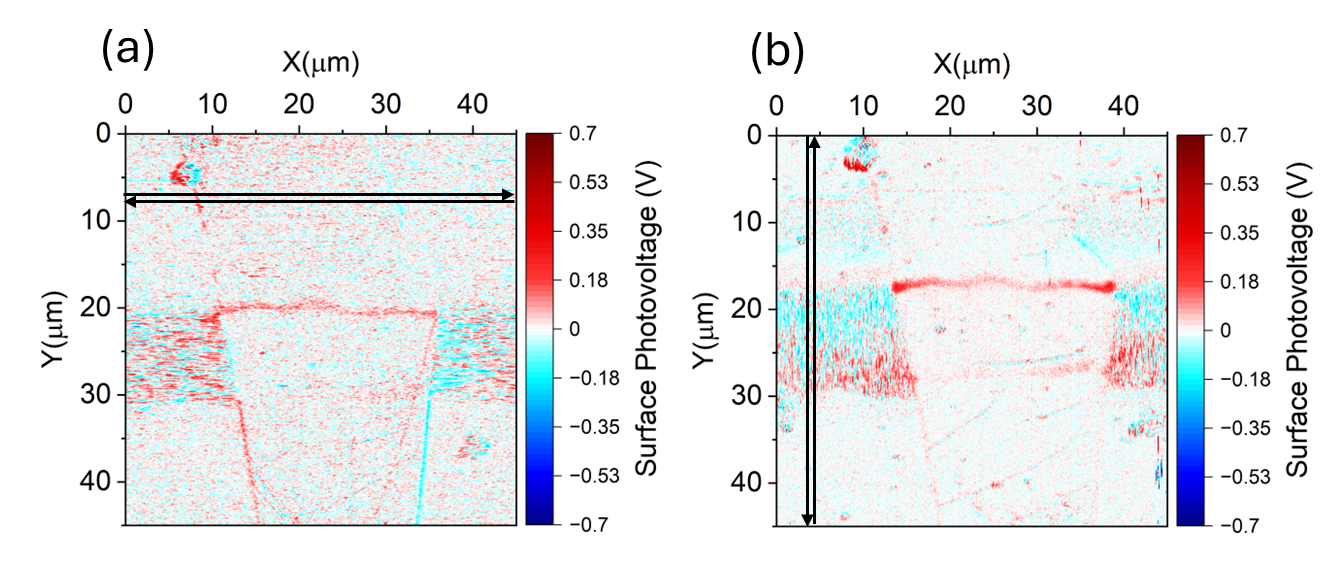


**FIG. S3.** SPV map of MoS_2_ flake on gold contacts scanning across the flake (a) and along the flake (b).

To investigate further, we adjusted the scan rates and found that the edge effects became more prominent with higher scan rates as presented in Fig.S4.

**
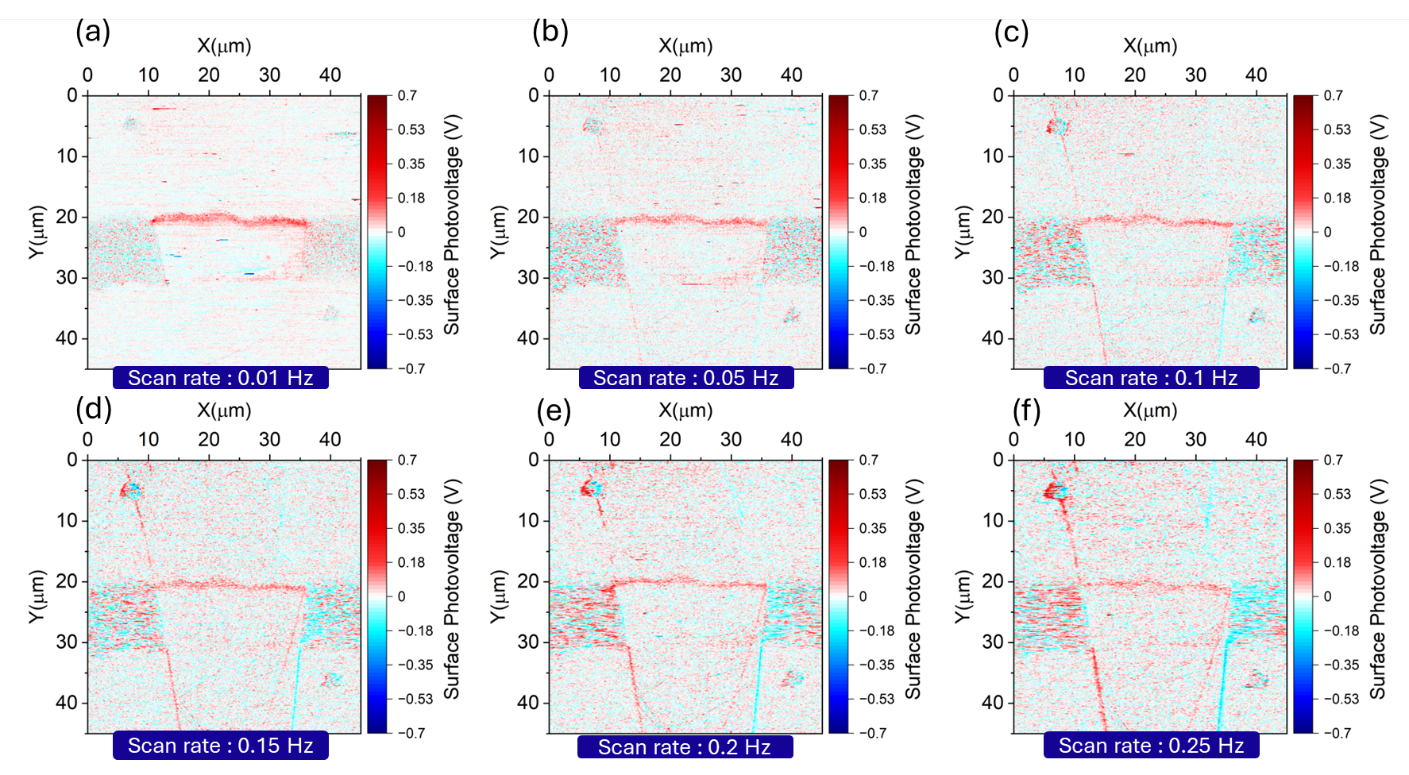
**

**FIG. S4.** SPV map derived from scan rates of 0.01 Hz (a), 0.05 Hz (b), 0.1 Hz (c), 0.15 Hz (d), 0.2 Hz (e) and 0.25 Hz (f).
